# Novel technique with the IRIS U kit to prevent urethral injury in patients undergoing transanal total mesorectal excision

**DOI:** 10.1016/j.amsu.2019.08.002

**Published:** 2019-08-13

**Authors:** Toshikatsu Nitta, Keitaro Tanaka, Jun Kataoka, Masato Ohta, Masatsugu Ishii, Takashi Ishibashi, Junji Okuda

**Affiliations:** a Division of Surgery Gastroenterological Center, Medico Shunju Shiroyama Hospital, Osaka, Japan; b Department of Colorectal Surgery Osaka Medical College, Osaka, Japan

**Keywords:** TaTME, IRIS U kit, Urethral injury, transanal total mesorectal excision, TaTME

## Abstract

**Background:**

Low anterior resection of the rectum with total mesorectal excision (TME) has been the gold standard for the surgical treatment of rectal cancer as it has the lowest recurrence rates. The key issue while performing transanal TME (TaTME) is avoiding iatrogenic urethral injury. We introduce our surgical technique for TaTME.

**Surgical technique:**

Intraurethral indocyanine green injection using the IRIS U kit with subsequent visualization under NIR was safely utilized during the TaTME. We were able to easily detect and visualize the IRIS urethral kit. The prostatic segment of the urethra can be identified in real-time using the infrared illumination system urethral kit (IRIS U kit).

**Benefits:**

The prostatic segment of the urethra was easily and quickly identified by the green fluorescence during TaTME.

**Conclusion:**

Our TaTME technique is an easy and feasible approach that provides real-time urethral images.

## Highlights

• New technique during TaTME.
• IRIS U-kit is device to avoid the urethra injury.

## 1 Introduction

Low anterior resection of the rectum with total mesorectal excision (TME) has been the gold standard for the surgical treatment of rectal cancer as it has the lowest recurrence rates [1]. Recently, transanal total mesorectal excision (TaTME) have been developed as options to retain the circumferential resection margins (CRM) during TME [2]. TaTME has become a popular method for the treatment of lower rectal cancer. However, navigating pelvic anatomy during TaTME can be a complicated process; surgeons often find it difficult to recognize the exact anatomy even if they have considerable experience of TaTME.

Urethral injury is a significant complication of TaTME because the dissection line of TaTME is close to the urethra. Some techniques [3,4] such as real time visualization of the ureters using indocyanine green have been reported to help avoid urethral injury. We have therefore introduced a new device, the IRIS U kit for transanal TaTME, to help surgeons avoid urethral injury.

## 2 Material and methods (surgical technique)

A total colonoscopy in a 58-year-old man revealed a lesion in the rectum below the peritoneal reflection (4 cm above the anal verge). The patient was subsequently scheduled for laparoscopic resection through the transanal approach for early rectal cancer. The patient was placed in the lithotomy position. A purse-string suture was placed approximately 1 cm from the anal verge and circumferential dissection was performed 1 cm from the purse-string suture. The AIRSEAL SYSTEM (SurgiQuest Inc, Milford, CT) was used to perform the TaTME after the GelPoint (Applied Medical Resources Corporation, Rancho Santa Margarita, CA) access system was set up.

The key issue while performing TaTME is avoiding iatrogenic urethral injury. This complication is sex-specific, because the urethra of male patients is close to the detachment plane of the rectum, especially in its lower urinary tract segments.

**Fig. 1:**
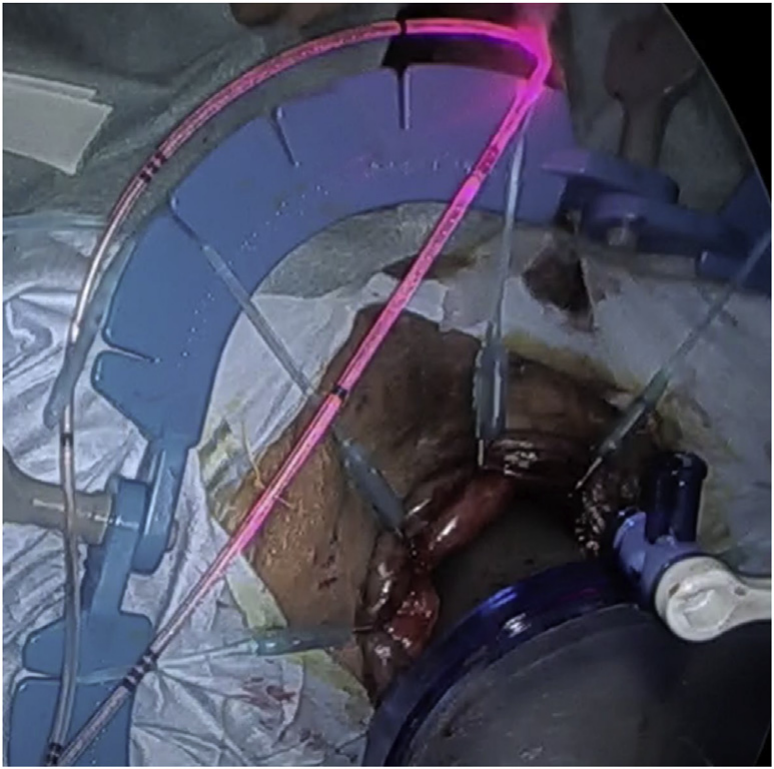
Infrared illumination system The pink light flashed on and off in real-time. . (For interpretation of the references to colour in this figure legend, the reader is referred to the Web version of this article.)

During TaTME in men, it is important to check the urethra, especially the prostatic segment. The anterior plane of the low rectum in men is immediately below the apex of the prostate and no clear landmarks exist. We can identify the prostatic segment of the urethra in real-time by using the infrared illumination system urethral kit (IRIS U kit) (Fig. 1) under endoscopic near-infrared (NIR) visualization.

Before the surgery, a urethral catheter with a guide wire was inserted into the urethra under fluoroscopic guidance, after which the IRIS U kit was used. Insertion of the IRIS urethral kit was performed by a urologist to guarantee safety.

Our hospital's institutional review board granted permission for use of the IRIS U kit. The NIR light was used to increase visibility when using the laparoscopic system (1588 AIM™; Stryker) during the laparoscopic and TaTME stages of the procedure.

**Fig. 2:**
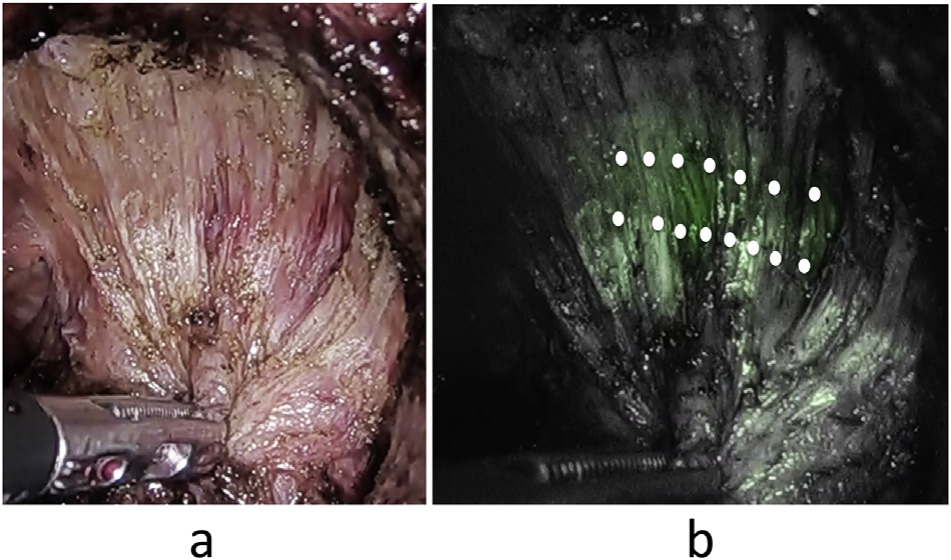
**a**: The prostatic urethra in the absence of near-infrared fluorescence. **b**: The prostatic urethra under near-infrared fluorescence. (double white dots).

We removed the IRIS U kit gradually until the urethra was separated from the ureter, and the anterior plane of the rectum immediately below the apex of the prostate became clearly visible (Fig. 2a, Fig. 2b). We were thus able to identify the urethra and perform conventional TaTME safely. This study was approved by the Ethics Committee of Shiroyama Hospital (2018–034) and registered with the Research Registry under the serial number 5000. The patient gave informed consent to participate in this study. This study is reported in accordance with the SCARE Guidelines [5].

## 3 Results

Intraurethral indocyanine green injection using the IRIS U kit with subsequent visualization under NIR was safely utilized during the TaTME.

We were able to easily detect and visualize the IRIS urethral kit. The prostatic segment of the urethra was easily and quickly identified by the green fluorescence during TaTME. The total operative time was 435 min, including a perineal phase of 122 min, and the intraoperative blood loss was 65 ml. The patient recovered uneventfully with no complications attributable to use of the IRIS urethral kit.

There were no complications attributable to IRIS U kit use.

### 3.1 The convalescence was unremarkable postoperatively

The total operative time was 435 min, including perineal phase time was 122 min and the intraoperative blood loss was 65 ml. The patient demonstrated a good postoperative course, and was discharged from our hospital in remission.

## 4 Discussion

TaTME was first reported in 2010 by Sylla et al. [6]. According to the International transanal Total Mesorectal Excision (TaTME) registry [2], TaTME appears to be an oncologically safe and effective technique for distal mesorectal dissection with acceptable short-term patient outcomes and good specimen quality. The registry data also showed a urethral injury rate of 1.0% (5/489), which is not higher than expected. It should be noted that this rate was achieved by international experts in TaTME. The highest urethral injury rate reported was 6.7%, which was recorded as part of a single-center study [7]. Urethral injury often occurs due to the unfamiliar view and the difficulty in identifying the correct tissue planes during TaTME, especially when operating on the prostatic urethra. Despite this being our first TaTME, although we were able to perform the procedure without urethral injury and the patient's recovery was uneventful.

TaTME operative time is recorded according to abdominal and perineal phases. The perineal phase constitutes the time from the purse-string suture to the anastomosis for TaTME. The International TaTME registry reported a mean total operative time of 277 ± 83 (62–685) min with a 128 ± 70 (15–467) min perineal phase. Our total operative time was 435 min, longer than previously reported, but the perineal phase was only 122 min. Our method enables good visualization of the urethra and dissection planes, enabling quick completion of the perineal phase.

Francis et al. [8] stated that overcoming the learning curve for TaTME required various training modalities and assessment. Studying this process also has the potential to improve technical quality and guide future research initiatives for this novel technique. There are several technical challenges involved in this technique, such as the unfamiliar view and interpretation of the anatomy from below, which may make it difficult to identify the correct tissue planes. They also stated that mentors should have performed at least 20 TaTME cases and be experienced in laparoscopic training. Accumulating experience in TaTME appears to be an important factor in avoiding urethral injury.

However, TaTME for low rectal cancer is not common because its indications are limited, and the number of cases performed to this day is limited. Our technique for TaTME is an easy and feasible method that provides real-time urethral images. Quick visualization of the urethra using the IRIS U kit enabled us to avoid urethral injury during our first transanal total mesorectal excision.

## Ethical approval

This case in not necessary to require ethical approval because this paper is just surgical technique however we got the Ethical approval for this study was obtained from ETHICS COMMITTEE Medico Shunjyu Shiroyama Hospital

(APPROVAL NUMBER/ID) SH 2018-034 by way of precaution.

## Sources of funding

No source of funding for this research.

Authors and all co-authors have no funding for your research.

## Author contribution

Toshikatsu Nitta is author:writing.

Keitaro Tanaka ans Junji Okuda: Help for operation and advise.

Kataoka Jun ans Ohta Masato and Masatsugu Ishii,Takashi Ishibashi: menmeber of this Surgery.

Thanks to them we performed this operation.

## Conflicts of interest

No conflict of interest to declare.

Authors and all co-authors have no conflicts of interest.

## Trial registry number

researchregistry5000.

https://www.researchregistry.com.

## Guarantor

All the authors of this paper accept full responsibility for the work and/or the conduct of the study, had access to the data, and controlled the decision to publish.

## Consent

Patient informed written consent was obtained andall identifying information is omitted.

This case in not necessary to require ethical approval because this paper is just surgical technique.

However we got the Ethical approval for this study was obtained from ETHICS COMMITTEE.

Medico Shunjyu Shiroyama Hospital.

(APPROVAL NUMBER/ID) SH 2018-034 by way of precaution.

## Provenance and peer review

Not commissioned, externally peer reviewed.

## Declaration of interest statement

None.
